# Connexin 43 hemichannels protect bone loss during estrogen deficiency

**DOI:** 10.1038/s41413-019-0050-2

**Published:** 2019-04-22

**Authors:** Liang Ma, Rui Hua, Yi Tian, Hongyun Cheng, Roberto Jose Fajardo, Joseph J. Pearson, Teja Guda, Daniel Brian Shropshire, Sumin Gu, Jean X. Jiang

**Affiliations:** 1 Department of Orthopaedics, Jinan Central Hospital Affiliated to Shandong University, Jinan, Shandong China; 2 Department of Biochemistry and Structural Biology, UT Health San Antonio, San Antonio, TX 78229 USA; 3 Department of Orthopedics, UT Health San Antonio, San Antonio, TX 78229 USA; 4 Department of Biomechanical Engineering, University of Texas at San Antonio, San Antonio, TX 78249 USA

**Keywords:** Bone quality and biomechanics, Bone

## Abstract

Estrogen deficiency in postmenopausal women is a major cause of bone loss, resulting in osteopenia, osteoporosis, and a high risk for bone fracture. Connexin 43 (Cx43) hemichannels (HCs) in osteocytes play an important role in osteocyte viability, bone formation, and remodeling. We showed here that estrogen deficiency reduced Cx43 expression and HC function. To determine if functional HCs protect osteocytes and bone loss during estrogen deficiency, we adopted an ovariectomy model in wild-type (WT) and two transgenic Cx43 mice: R76W (dominant-negative mutant inhibiting only gap junction channels) and Cx43 Δ130–136 (dominant-negative mutant compromising both gap junction channels and HCs). The bone mineral density (BMD), bone structure, and histomorphometric changes of cortical and trabecular bones after ovariectomy were investigated. Our results showed that the Δ130–136 transgenic cohort had greatly decreased vertebral trabecular bone mass compared to WT and R76W mice, associated with a significant increase in the number of apoptotic osteocyte and empty lacunae. Moreover, osteoclast surfaces in trabecular and cortical bones were increased after ovariectomy in the R76W and WT mice, respectively, but not in ∆130–136 mice. These data demonstrate that impairment of Cx43 HCs in osteocytes accelerates vertebral trabecular bone loss and increase in osteocyte apoptosis, and further suggest that Cx43 HCs in osteocytes protect trabecular bone against catabolic effects due to estrogen deficiency.

## Osteoporosis: Hemichannels protect bone from estrogen deficiency

Channels that form between cells and their extracellular environment help protect bone tissue from the damage wrought by low estrogen levels, a major cause of bone loss in post-menopausal women. Jean Jiang from the UT Health San Antonio and colleagues showed that depleting the estrogen hormone in mouse bone cells reduced levels of connexin 43 and impaired the protein’s ability to forms pores known as ‘hemichannels’. The researchers surgically removed the ovaries of various mouse strains to induce estrogen deficiencies. They found that transgenic mice without working hemichannels had reduced bone mass compared to normal mice or mice that could make hemichannels but lacked the ability for those channels to come together to form complete passageways. The findings highlight the importance of connexin 43 hemichannels in protecting bone tissue against osteoporosis.

## Introduction

Osteoporosis and osteopenia, diseases of low bone mineral density (BMD), are common among postmenopausal women and feature a high risk of fragility fracture.^1^ Decreased estrogen is closely associated with an increase in osteoclast-mediated bone resorption and a decrease in osteoblast-mediated bone formation, which contribute to bone loss during aging.^2–6^ Furthermore, estrogen deficiency, oxidative stress (OS), micro-damage, and aging influence the viability of osteocytes, which are required for mediating the efficient bone remodeling, quality maintenance, and damage repair processes of bone.^7–9^

Osteocytes, comprising about 95% of bone cells, are embedded within the bone matrix and communicate with neighboring osteocytes and cells on the bone surface (BS) through an extensive network of long dendritic processes.^10^ Gap junctions are intercellular channels that provide direct cell–cell communication between adjacent cells. Connexin 43 (Cx43) forms gap junctions that mediate osteocyte coupling and are critical for maintaining proper bone physiology, including proliferation, survival and differentiation of osteoblasts, and skeletal development.^11–13^ Hemichannels (HCs), unpaired gap junction channels, are extensively involved in the communication between osteocytes and their extracellular environment, and play important roles in autocrine/paracrine signaling, cell survival, and mechanotransduction.^14–17^ Connexin-based gap junctions and HCs allow the passage of small molecules (MW < 1 000 Da), such as ions, essential metabolites, and second messengers, such as Ca^2+^, IP3, NAD^+^, prostaglandin E_2_ (PGE_2_), cAMP, cGMP, ADP, and ATP.

In recent years, the importance of Cx43 in bone formation, remodeling, and responses to mechanical loading and parathyroid hormone has been illustrated using osteoblast- and osteocyte-specific conditional Cx43 knockout (cKO) mouse models.^18–23^ Moreover, deficiency of Cx43 primarily in osteocytes shows increased apoptosis associated with empty lacunae.^24^ However, these gene KO models could not elucidate the specific role of connexin channels given that Cx43 forms both gap junction channels and HCs. We generated two transgenic mouse models driven by DMP1 promoter with the expression of Cx43 mutants predominantly in osteocytes: R76W, with dominant-negative effects on gap junction channels; and Δ130–136, with dominant-negative effects on both gap junction channels and HCs.^25^ We showed that impairment of Cx43 HCs negatively affected bone formation, remodeling, and osteocyte viability. These two transgenic mouse models allow us to dissect differential functions of gap junctions vs. HCs formed by Cx43.

To study the specific role of osteocyte Cx43 channels in estrogen-deficient bone tissue, we conducted ovariectomies on wild-type (WT) and two transgenic Cx43 mouse strains, R76W and Δ130–136, and then determined bone structure, bone histomorphometry, osteocyte viability, and bone markers of these ovariectomized (OVX) mouse lines. By using various histochemical and bone histomorphometry approaches in cortical and trabecular bones, we show that Cx43 HCs play an important role in osteocyte viability, bone remodeling, and OS-related lipid peroxidation under mouse models of estrogen deficiency-induced osteoporosis.

## Results

### Estrogen deficiency reduced Cx43 expression and HC function

**Fig. 1:**
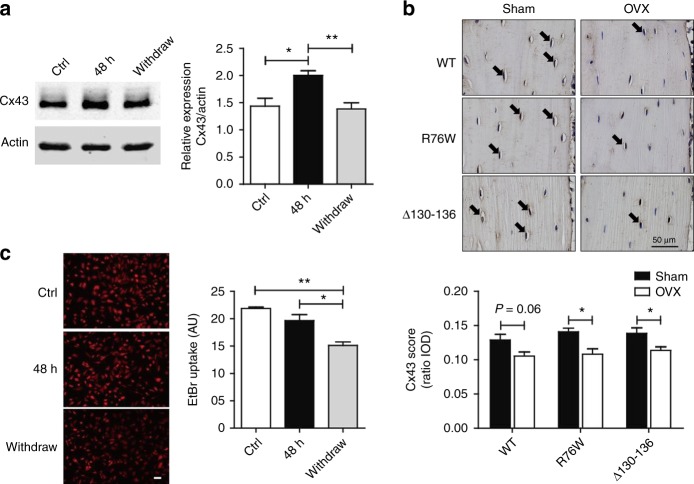
Estrogen deficiency reduced connexin 43 (Cx43) expression and hemichannel function. **a** Membrane extracts were subjected to immunoblotting using anti-Cx43 (CT) or β-actin antibodies. The right panel shows the densitometric measurement ratios of Cx43 to β-actin (*n* = 5). **b** Paraffin sections of femoral cortical bone were immunolabeled with Cx43 (CT) antibody followed by incubation with avidin–biotin–peroxidase complex (ABC) reagent. Immunohistochemistry staining showed lower levels of Cx43 expression in ovariectomized mice (brown signals, solid arrows, upper panel). The Cx43 score was determined as a ratio of integrated optical density (IOD) to cortical bone area (lower panel, *n* = 3–6). **c** Hemichannel dye uptake was performed with ethidium bromide (EtBr) (red fluorescence). Left panels showed representative fluorescence images of treated MLO-Y4 cells after dye uptake. The intensity of EtBr fluorescence was measured and quantified (right panel). Data shown are mean ± SEM. *P < 0.05; **P < 0.01

17β-Estradiol is a potent naturally circulating endogenous estrogen.^26^ To determine the effects of estrogen deficiency on Cx43 expression in osteocytes, we first treated MLO-Y4 cells with 100 nmol·L^-1^ 17β-estradiol for 24 h, and estrogen was then either maintained or withdrawn from the culture medium for another 24 h. The 48 h estrogen treatment increased Cx43 expression detected by Western blotting, while compared to the estrogen treatment group, the expression of Cx43 decreased significantly in the estrogen withdrawal group (Fig. 1a). We also examined the expression of osteocytic Cx43 in bone sections from OVX and sham mice by immunohistochemistry. The expression pattern of Cx43 was similar with our previous published paper.^7^ The results showed that positive staining of Cx43 protein was less visible in the osteocytes of the OVX group as opposed to the sham group (Fig. 1b, upper panel, arrowheads). Data quantification showed a significant decrease of osteocytic Cx43 expression in OVX mice compared to sham mice (Fig. 1b, lower panel). The activities of Cx43 HCs in response to estrogen withdrawal were further studied using ethidium bromide (EtBr) dye uptake assay. Data analysis showed that opening of HCs detected by the uptake of the dye was significantly reduced by estrogen withdrawal as compared to 48 h estrogen-treated MLO-Y4 cells (Fig. 1c). In addition, the effect of estrogen treatment for only 24 h was also examined. Cx43 expression increased significantly compared to control (Fig. S1A), while the Etbr dye uptake remained the same between treated and non-treated groups (Fig. S1B). These results suggest that estrogen withdrawal decreased Cx43 expression and HC function in osteocytes.

### Acceleration of bone loss and alteration of bone phenotypes with the impairment of osteocytic HCs in OVX mice

**Fig. 2:**
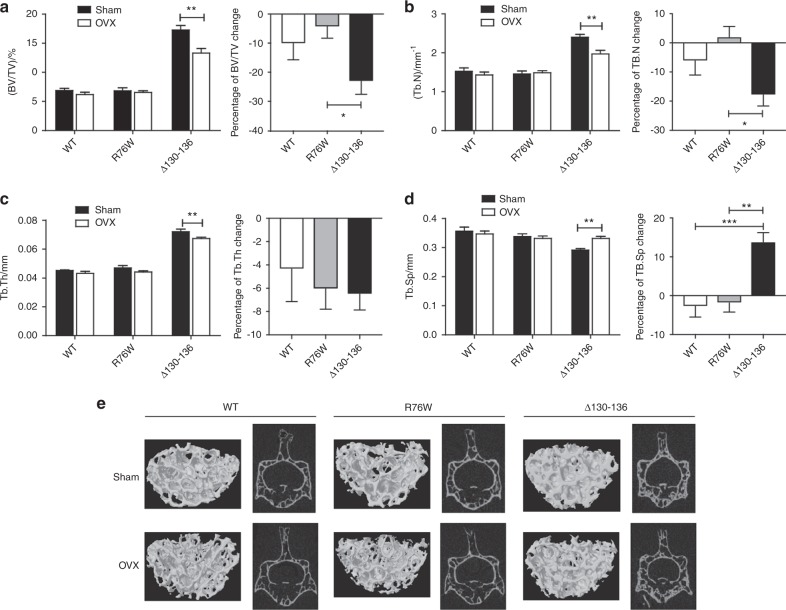
Increased bone loss and altered structure of vertebral trabecular bones in Δ130–136 mice after ovariectomy. Three-dimensional micro-computed tomography (3D µCT) analysis of BV/TV (**a**), Tb.N (**b**), Tb.Th (**c**), and Tb.Sp (**d**) of fifth lumbar (L5) vertebral trabecular bone showed significantly increased bone loss in Δ130–136 mice compared to wild-type (WT) and R76W mice. Left panels show the corresponding percentage changes. Representative 3D µCT images of L5 vertebra of WT and transgenic groups were shown (**e**). Data shown are mean ± SEM. *P < 0.05; **P < 0.01; ***P < 0.001. *n* = 6–7. BV/TV, bone volume fraction; Tb.N, trabecular number; Tb.Th, trabecular thickness; Tb.Sp, trabecular spacing

As reported previously,^27^ at 4 weeks after surgery, the body weight of OVX mice was increased compared to sham-operated counterparts, along with a dramatically decreased size and weight of uterus (Fig. S2A). In addition, OVX mice showed a significant decrease in whole-body BMD in WT and transgenic mice compared to sham-operated counterparts (Fig. S2B). The bone geometry was assessed by three-dimensional micro-computed tomography (3-D μCT) imaging system. Data from μCT analysis of fifth lumbar vertebrae (L5) showed that after ovariectomy the trabecular bone volume fraction (BV/TV, Fig. 2a), number (Tb.N, Fig. 2b), and thickness (Tb.Th, Fig. 2c) were significantly reduced associated with a significant increase in trabecular separation (Tb.Sp, Fig. 2d) in the ∆130–136 OVX group. These significant alterations were not observed in WT and R76W OVX groups compared to sham-operated counterparts. In comparing among groups with the percentage changes, the BV/TV (Fig. 2a, right panel) and Tb.N (Fig. 2b, right panel) of Δ130–136 mice were significantly reduced, although there was no significant difference in Tb.Th (Fig. 2c, right panel). Consistently, the Tb.Sp percentage change (Fig. 2d, right panel) was significantly increased compared to WT and R76W groups. Similarly, we analyzed trabecular bone from distal femur. Contrary to vertebral bone response, there was no significant alteration of bone mass and other trabecular structure properties in Δ130–136 mice compared to WT and R76W mice (Fig. S3). These data show that Δ130–136 mouse augments bone loss in vertebral trabecular bone after ovariectomy.

**Fig. 3:**
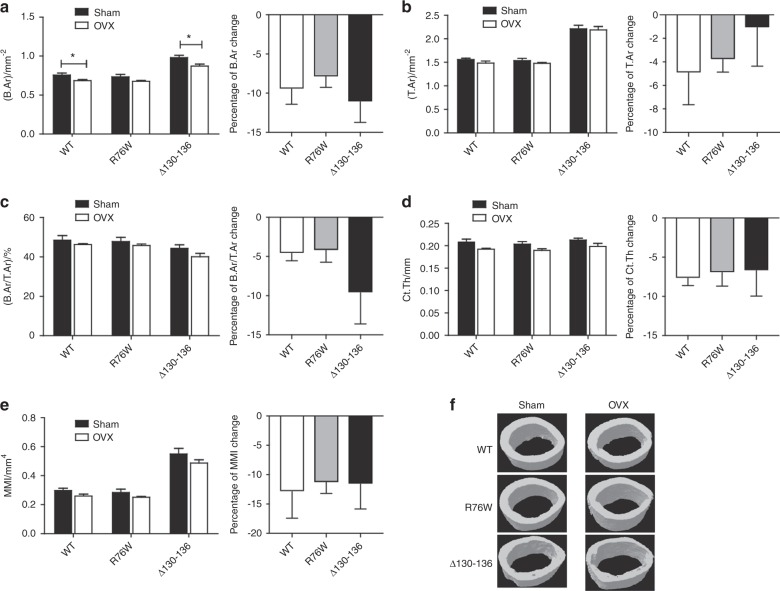
Minimal alterations of cortical bone properties in Δ130–136 mice after ovariectomy. Three-dimensional micro-computed tomography (3D µCT) analysis of B.Ar (**a**), T.Ar (**b**), B.Ar/T.Ar (**c**), Ct.Th (**d**), and MMI (**e**) of femoral midshaft cortical bone were shown. **f** Representative µCT images of cross-sections images of femoral cortical bone. Data shown are mean ± SEM. *P < 0.05. *n* = 6–7. T.Ar, total area; B.Ar, bone area; Ct.Th, cortical thickness; MMI, area moment of inertia

We evaluated femoral cortical bone properties after ovariectomy by μCT (Fig. 3). The data from cortical bone showed that the bone area (B.Ar) of the femur midshaft after ovariectomy in WT and ∆130–136 groups was significantly reduced compared to corresponding sham-operated groups (Fig. 3a), while tissue area (T.Ar) was similar between groups (Fig. 3b). Values for B.Ar/T.Ar (Fig. 3c), cortical thickness (Ct.Th, Fig. 3d), and area moment of inertia (MMI, Fig. 3e) were not significantly changed between sham and OVX groups. However, there were no significant differences in the percentage change of any group (Fig. 3, corresponding right panels). Roughened surface of cortical bone at the femur diaphysis was observed by µCT imaging of the cross-sections of midshaft bone in all OVX groups, with more severe endocortical resorption shown in OVX Δ130–136 mice (Fig. 3f). The mean total cross-sectional marrow area of Δ130–136 mice is the largest compared to WT and R76W and ovariectomy does not affect the marrow areas in WT and two transgenic models compared to corresponding sham controls (Fig S4). Together, these data suggest that ovariectomy leads to significantly more vertebral trabecular bone loss in ∆130–136 than WT and R76W mice, while there are no significant differences of cortical bone loss and marrow areas among all three genotypes.

**Fig. 4:**
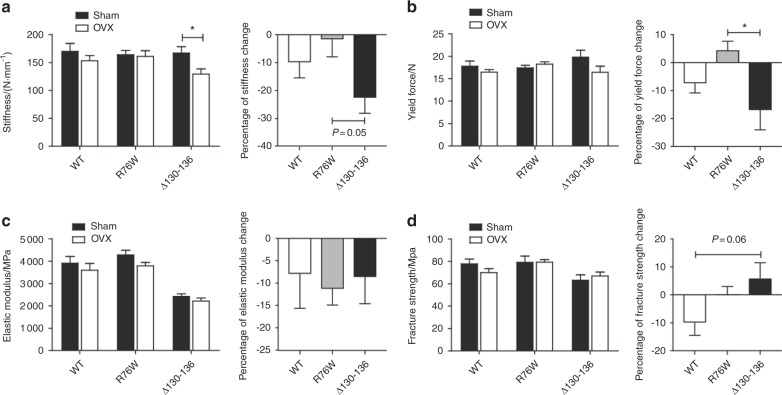
Increased deterioration of bone material properties in Δ130–136 mice after ovariectomy. Three-point bending assay was performed on isolate femur bones of wild-type (WT) and transgenic mice, and stiffness (**a**), yield force (**b**), elastic modulus (**c**), and fracture strength (**d**) were determined. Stiffness and yield force were reduced in ovariectomized Δ130–136, while elastic modulus and fracture strength were not altered or increased, respectively. Data shown are mean ± SEM. *P < 0.05. *n* = 6

Biomechanical properties of the bone were analyzed using three-point bending flexural evaluation of the femurs. Ovariectomy significantly reduced bone stiffness in ∆130–136 mice, while no significant changes were observed in WT and R76W groups (Fig. 4a). The percentage of stiffness change further showed a close-to-significant decrease (Fig. 4a, right panel, *P* = 0.05). There was a significant decrease shown in the percentage of yield force change in the ∆130–136 and R76W groups (Fig. 4b, right panel). Interestingly, OVX ∆130–136 mice had similar material properties of elastic modulus (Fig. 4c) as the WT and R76W mice. However, the percentage of fracture strength change showed a trend of elevation in ∆130–136 compared to WT group (*P* = 0.06), although there was no apparent difference between sham and OVX in WT and transgenic groups (Fig. 4d). Together, given the functional differences of R76W and Δ130–136 on gap junctions and gap junctions/HCs, respectively, these data indicate that HCs are likely to protect bone loss and bone fragility during estrogen deficiency.

### Increased cortical osteocyte apoptosis and empty lacunae in OVX ∆130–136 mice

**Fig. 5:**
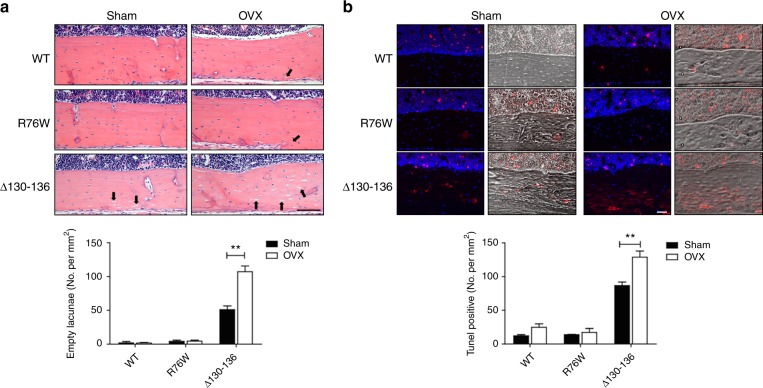
Significant increase of apoptotic osteocytes and empty lacunae in ∆130–136 mice after ovariectomy. **a** Hematoxylin and eosin (H&E) staining of paraffin sections showed increased numbers of empty lacunae in femoral cortical bones in ovariectomized Δ130–136 mice, but minimal differences in R76W compared to WT mice. The solid arrowheads point to the empty lacunae. Scale bar = 200 μm. **b** Terminal deoxynucleotidyl transferase (TdT) dUTP nick-end labeling (TUNEL) staining showed increased signals (red) in osteocytes of ovariectomized Δ130–136 mice; the nuclei counter-stained with 4′,6-diamidino-2-phenylindole (DAPI) (blue) (left panels) and merged phase images (right panels). Scale bar = 100 μm. Empty lacunae and TUNEL signals per mm^2^ of cortical bone area were quantified by NIH Image J. Data shown are mean ± SEM. **P < 0.01. *n* = 4–7

Hematoxylin and eosin (H&E)-stained cortical bone sections from femur were used to assess osteocytic lacunae (Fig. 5a). We have previously shown that there are more empty lacunae in cortical bones of Δ130–136 mice than WT and R76W mice.^25^ Quantification showed that ovariectomy increased numbers of empty lacunae in Δ130–136 almost two-fold, but this increase was not observed in WT and R76W mice (Fig. 5a, lower panel). Noticeably, the disorganized matrix structure of Δ130–136 mice cortical bones was indicated by lightly, uneven staining by H&E in sham and OVX mice.

Consistent with the observation of increased numbers of empty lacunae by ovariectomy, TUNEL staining revealed a significant increase in apoptotic osteocytes in cortical bones of Δ130–136 OVX mice (Fig. 5b). The presence of TUNEL labeling (red) was minimal in sham mice. We also analyzed the TUNEL staining in tibia trabecular region and showed that ∆130–136 OVX mice exhibited a trend of increased osteocyte apoptosis compared to sham (*P* = 0.07) (Fig. S5). Together, these data show that osteocytes in the cortical bone of ∆130–136 were more vulnerable to ovariectomy-induced cell death than those in WT and R76W mice, and further suggest that Cx43 HCs protect osteocytes and prevent bone loss caused by estrogen deficiency.

### Attenuation of ovariectomy-induced osteoclast activation with impairment of HCs

**Fig. 6:**
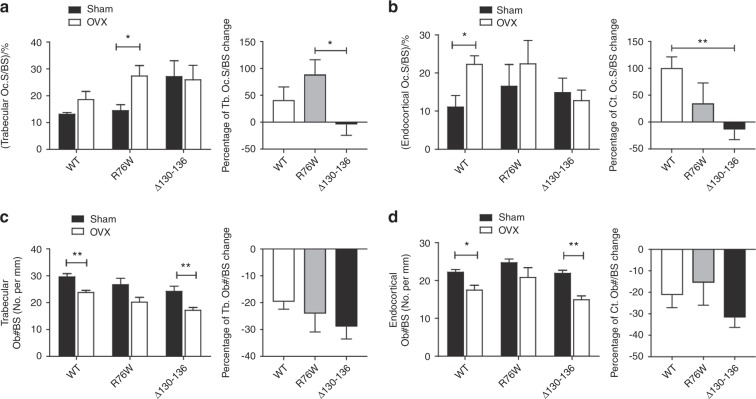
Reduction of Oc.S changes in trabecular and cortical bones of ∆130–136 mice after ovariectomy. **a**, **b** Paraffin sections of femoral bone were stained by TRAP and osteoclasts were labeled and quantified. Femoral trabecular and endocortical Oc.S was increased in wild-type (WT) and R76W mice after ovariectomy, but not in Δ130–136 transgenic mice. **c**, **d** Paraffin sections of femoral bone were stained with Masson’s trichrome, and osteoblasts were labeled and quantified. Data shown are mean ± SEM. *P < 0.05; **P < 0.01. *n* = 6–7. OB#, number of osteoblasts; BS, bone surface; OC.S, osteoclast surface; TRAP, tartrate-resistant acid phosphatase

The trabecular osteoclast surface to BS ratio (Oc.S/BS) in R76W mice was significantly increased (Fig. 6a), while endocortical Oc.S/BS was significantly increased in WT (Fig. 6b) compared with their sham-operated counterparts. However, this increase was blunted in ∆130–136 mice. The attenuation of this effect was further shown by the percentage of Oc.S/BS change (Fig. 6a, b, right panels). The percentage change of Oc.S/BS values in trabecular and endocortical bones of ∆130–136 group were significantly lower compared to R76W and WT mice groups, respectively. These data suggest that Cx43 HCs in osteocytes influence the formation of osteoclasts during estrogen deficiency-induced bone loss.

**Fig. 7:**
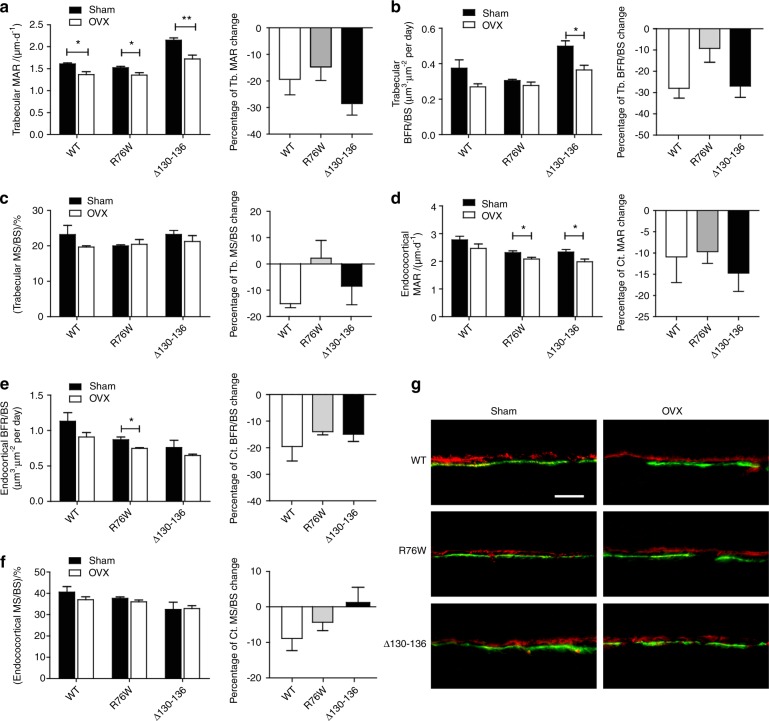
Minimal changes of bone dynamic histomorphometry parameters in ovariectomized Δ130–136 transgenic mice compared with wild-type (WT) and R76W groups. Mice were injected twice with calcein and alizarin dyes, and plastic sections were prepared. Trabecular (**a–c**) and cortical (**d–f**) MAR, BFR/BS, and MS/BS were measured in unstained sections from the tibial mid-diaphysis. Representative images were shown in **g**. Scale bar = 100 μm. Data shown are mean ± SEM. *P < 0.05; **P < 0.01. *n* = 4. MAR, mineral apposition rate; BFR, bone formation rate; BS, bone surface; MS, mineralizing surface

Consistent with previous reports,^28,29^ we showed that ovariectomy reduced osteoblast numbers in both trabecular and endocortical bones in WT mice (Fig. 6c, d). A similar reduction was also observed in ∆130–136 mice. Unlike the effect on osteoclasts, the percentage of osteoblast changes showed no significant difference between WT and transgenic groups (Fig. 6c, d, right panels). We analyzed the dynamic bone histomorphometry parameters using a calcein and alizarin double labeling assay. Consistent with previous studies,^30^ ovariectomy decreased the mineral apposition rate (MAR) of trabecular (Fig. 7a, left panel) bones in WT mice. The trabecular and cortical bone formation rate per BS (BFR/BS) were significantly lower in the ∆130–136 and R76W OVX groups, compared to the sham groups, respectively (Fig. 7b–e, left panels). No differences in the percent changes of other bone formation parameters, mineralizing surface per BS (MS/BS) or BFR/BS, were detected between WT and two transgenic groups (Fig. 7b–f, right panels). Together, the dynamic histomorphometry showed the change of bone formation to some extent in WT and both transgenic models; however, the difference shown in cortical BFR/BS or MAR alone showed similar trend of reduction, but with significant difference shown in R76W in Fig. 7e, and there were no differences found in other parameters. Based on these data, the involvement of gap junctions and HCs is less apparent in bone formation. These data suggest that impairment of gap junctions and/or HCs has minimal impacts on osteoblast numbers and bone formation in response to ovariectomy.

### Changes of OS and bone markers in transgenic Cx43 OVX mice

**Fig. 8:**
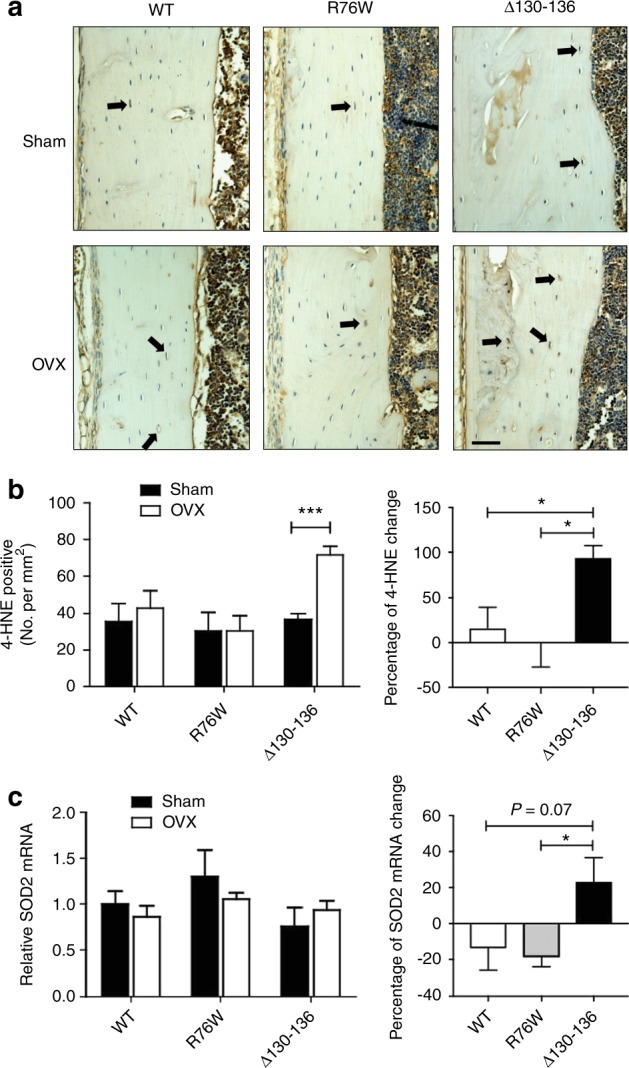
Increased oxidative stress level in ∆130–136 mice after ovariectomy. **a** Paraffin sections of femoral cortical bone were immunolabeled with anti-4-hydroxynoneanal (4-HNE) antibody (brown signals). Immunohistochemistry staining showed higher level of 4-HNE signals in ovariectomized Δ130–136 mice (solid arrows). Scale bar = 100 μm. **b** 4-HNE-positive osteocytes per mm^2^ of cortical bone area were quantified. **c** Real-time quantitative reverse transcription-PCR (qRT-PCR) was performed with RNAs extracted from bone tissues. The percentage of superoxide dismutase 2 (SOD2) messenger RNA (mRNA) change was elevated in Δ130–136 compared to wild-type (WT) and R76W mice. Data shown are mean ± SEM. *P < 0.05; ***P < 0.001. *n* = 3–5

Estrogen deficiency elevates OS levels in OVX bone tissues.^31^ The above data show that impaired HCs augmented cell death and bone loss induced by ovariectomy. To determine if HCs have any effect on increase of OS levels in response to ovariectomy, we determined the level of 4-hydroxynoneanal (4-HNE), a biomarker for lipid peroxidation and OS by immunohistochemistry. Increased dense staining (black arrow) was observed in the ∆130–136 compared to WT and R76W OVX mice (Fig. 8a). Quantification of 4-HNE-positive cells per mm^2^ showed that Δ130–136 OVX mice had almost a two-fold increase compared to sham control and this increase was not observed in R76W and WT OVX mice (Fig. 8b, left panel). The percentage of change further confirmed the increase of 4-HNE levels by ovariectomy in ∆130–136 group (Fig. 8b, right panel). The evidence of elevated OS in Δ130–136 mice was further supported by increased amounts of superoxide dismutase 2 (SOD2) (Fig. 8c). Real-time quantitative reverse transcription-PCR (qRT-PCR) showed that SOD2 in ∆130–136 was elevated, different from WT and R76W by ovariectomy as the latter showed slight reduction. These results suggest that ablation of HCs renders osteocytes more vulnerable to OS after ovariectomy.

## Discussion

In this study, we found that bone loss and defects in osteocyte-specific Cx43 ∆130–136 OVX mice are more evident compared to R76W and WT mice, which includes increased vertebral trabecular bone loss, osteocyte apoptosis, compromised bone material quality, and increased OS levels. Since ∆130–136 has a dominant-negative effect on both gap junction channels and HCs, while R76W only on gap junction channels, our results suggest that impairment of Cx43 HCs in osteocytes augments the catabolic effect of ovariectomy on bone structure and quality. Therefore, osteocytic Cx43 HCs are likely to play a cell-protective role against trabecular bone loss and comprised bone quality due to postmenopausal estrogen deficiency and aging.

Previous studies by our group have shown that bones of ∆130–136 male mice have higher BMD and larger bone marrow cavities than those of WT and R76W, partially due to increased endocortical bone resorption coupled with increased periosteal bone formation.^25^ Despite the increased size of cortical bones, ∆130–136 mice exhibit increased apoptotic osteocytes associated with decreased B.Ars and compromised material properties. In the current study, we observed similar alterations of bone mass, structures, and material properties in female ∆130–136 mice. These structural and bone material changes caused by impaired HCs in either sex could make the bone more vulnerable under adverse conditions such as OS and aging.

∆130–136 mice share several similar features to Cx43 KO mouse models generated by osteocyte-specific DMP1-Cre, including enlarged bone marrow cavity, increased osteocytic apoptosis, and compromised material properties.^24,32^ However, in contrast to femoral trabecular bone, vertebral trabecular bone mass was greatly increased in ∆130–136 mice. Previous studies using Cx43 KO models primarily examine trabecular bones from femur and tibia; however, a study by Pacheco-Costa et al.^33^ showed that Cx43 KO by DMP1 Cre did not alter cancellous BV. We believe that animal models we used could cause this difference. Cx43 KO and Δ130–136 are two different models. Knocking out Cx43 ablates the function of both types of channels with the possibility of altered expression of other connexins/proteins. Our transgenic models with overexpression of dominant-negative mutants offer unique opportunity to dissect the specific involvement of these two types of channels by Cx43. The increased bone mass in vertebrae possibly attributes to the function of Cx43 HCs. In order to compare the OVX-induced bone loss among WT, R76W, and ∆130–136 mice, we normalized the OVX group with its corresponding control to obtain the ratio, thus reflecting the percent change regardless of the basal levels.

Ovariectomy in mice has been extensively used to model estrogen deficiency postmenopausal process in humans. Additionally, this model elevates OS levels in multiple organs including bone,^34–36^ which mimics the normal aging process. Previous studies showed that the magnitude of bone loss of OVX mice is influenced by genetic background determined by longitudinal μCT. Among the different inbred strains, C57BL/6J mice have much lower BV/TV at baseline, and there is no significant proximal tibia trabecular bone loss observed in C57BL/6J OVX mice compared to sham mice during the 5-week period.^37^ Li et al.^38^ reported that the alterations of cortical bone parameters occur 16 weeks after OVX for C57BL/6J mice, relatively slow compared to 4 weeks for C3H, and 8 to 16 weeks for A/J mouse strains. In our study, we used C57BL/6J mice and examined bone phenotypes at 4 weeks after surgery. Consistent with previous published studies in WT mice,^28,39,40^ we showed that at 4 weeks after ovariectomy surgery there was a reduction of total BMD along with alterations in BV (Fig. S3) and B.Ar (Fig. 3a) in femoral trabecular and cortical bones, respectively. The other bone phenotypes in WT mice in our study are also comparable with the previous reported studies.^41,42^ The success of ovariectomy experiment was evaluated by comparing the size and weight of uteruses. Uterine wet weight of the WT OVX mice was markedly lower than that of the intact controls (Fig. S2). Interestingly, we observed significant changes in osteocyte apoptosis, vertebral trabecular bone structures, and material properties in ∆130–136 mice compared to WT and R76W.

Our μCT analysis indicated that ovariectomy had different impacts on trabecular vs. cortical bone in ∆130–136 mice, with stronger catabolic effects on vertebral trabecular bones than on cortical bones. However, this difference was not observed in femoral trabecular bones, which is consistent with a published study in an osteoblastic Cx43 conditional KO model driven by 2.3 kb *Col1α1* promoter.^43^ Our study was conducted 4 weeks after ovariectomy. With this relative short time duration, we could not exclude the possibility that cortical bone phenotypes might appear at a later period after the surgery, considering the presence of large numbers of empty lacunae and apoptotic osteocytes in OVX ∆130–136 mice. Another possible explanation for the disconnection between cell numbers and structural changes is that the histomorphometry is 2D measurement, while the μCT characterizes overall bone architecture in 3D dimension. Despite the certain correlations between these two methods, previous studies also reported some discordant results with these two different methods.^44,45^ In addition, in support of our observation, clinical studies report that the trabecular bone is more vulnerable to bone loss associated with a higher risk of fractures than the cortical bone in osteoporotic patients.^46,47^ Moreover, vertebral fracture is more common in postmenopausal women, and treatment with an estrogen-related drug, raloxifene, greatly increases BMD and reduces risk of vertebral fracture.^48^ However, recent clinical studies suggest that, in contrast to the cortical bone, trabecular bone loss can be estrogen independent in humans.^2^ These studies point to complex mechanisms in controlling bone mass and imply the likely involvement of more than one key component.

Estrogen attenuates osteocyte apoptosis mediated by the ligand-binding domain of the receptor protein, involving activation of a Src/Shc/ERK signaling pathway.^49^ This process requires kinase-dependent activation of transcription factors and nuclear accumulation of ERKs.^50,51^ Our previous work demonstrated that extracellular PGE_2_ is responsible for the activation of p44/42 ERK signaling and Cx43 phosphorylation.^52^ In addition, Ren et al.^53^ reported that estrogen up-regulated Cx43 expression and enhanced gap junction intercellular communication in osteocyte-like MLO-Y4 cells. The aforementioned studies indicated a complex regulation mechanism of estrogen on osteocyte apoptosis and its relationship with Cx43. The estrogen deficiency-induced osteocyte apoptosis was reported in human bone biopsy samples^54^ and in rodent ovariectomy models.^40,55,56^ Studies by Emerton et al.^55^ showed that apoptotic osteocytes were significantly increased in the posterior femoral cortical regions after ovariectomy, but not elsewhere in the cortex, indicating that osteocyte apoptosis following estrogen loss occurs regionally. Another study using rat ovariectomy model reported a significant reduction in the percentage of apoptotic osteocytes associated with increasing distance from the growth plate in the cortical bone.^56^ Consistently, we showed a trend of increased apoptotic osteocytes in femoral cortical bone in WT OVX mice compared to the sham group. However, the difference did not reach significant level (*P* = 0.08), possibly due to the regions we quantified, consisting of both anterior and posterior of the mid-diaphysis.

We showed augmentation of osteocyte cell apoptosis and death as evidenced by empty lacunae and increased apoptotic signals in OVX ∆130–136 mice. Osteocyte survival plays a key role in normal bone homeostasis.^57^ The involvement of Cx43 protein has been suggested; mice deficient of Cx43 in osteocytes show increased bone marrow cavity area and increased cortical area, along with increased osteocyte apoptosis in cortical bone and impaired bone material properties.^24,32^ However, the KO models that ablate the expression of Cx43 could not distinguish the specific roles of gap junction channels vs. HCs. We and others have previously shown in vitro that Cx43 HCs protect cells against osteocyte apoptosis and cell death. An earlier study by Plotkin et al.^58^ showed that the anti-apoptotic role of bisphosphonates, drugs used to treat osteoporosis and osteopenia, on osteocytes is mediated through the activation of Cx43 HCs. We also showed that inhibition of HCs by anti-Cx43 antibody in osteocytes exacerbates H_2_O_2_-induced osteocytic cell death.^7^ This prior in vitro evidence points to an osteocyte-protective function of Cx43 HCs. In this study, through the ovariectomy model, we demonstrate a critical role of osteocytic Cx43 HCs in vivo in preserving cell survival against oxidative damage caused by estrogen deficiency. The increased osteocytic apoptosis and cell death are likely caused by elevated OS. Indeed, elevated OS, as indicated by increased levels of lipid peroxidation by 4-HNE and a reactive oxygen clearance enzyme SOD2, was evidenced in the OVX ∆130–136 model.

Noticeably, TUNEL-positive staining was detected in bone marrow cells of WT, R76W, and ∆130–136 mice, which is consistent with our previously published paper,^59^ and several reports from other groups.^23,60,61^ A possible interpretation is that bone marrow is the primary site of hematopoiesis. Hematopoietic stem cells (HSCs) undergo self-renewal or differentiation process. There is a large overcapacity of differentiating hematopoietic cells, and apoptosis is an important mechanism for regulating HSC numbers and homeostasis. Domen et al.^62^ employed a hematopoietic BCL-2 overexpression mouse model to investigate the role of apoptosis in the regulation of HSC numbers in vivo. H2K-*BCL-2* transgenic mice have increased numbers of HSC in bone marrow, along with affected cell cycle status. Another study showed that microRNA-146a induces lineage-negative bone marrow cell apoptosis by suppressing polo-like kinase 2 expression.^63^

It is interesting that the increase in osteoclast activity on endocortical BS after ovariectomy shown in WT and R76W was blunted in ∆130–136 mice. It is possible that apoptosis of OC/OC progenitors is affected during this process and estrogen treatment has been proved to promote OC apoptosis in vitro and in sham/OVX mice in vivo.^64,65^ Alternatively, as a master orchestrator of bone, osteocytes produce cytokines that regulate osteoclast formation and survival, and there is an association between osteocyte apoptosis and osteoclast recruitment in response to OVX.^55^ A similar observation was reported by Watkins et al.^43^ showing that ovariectomy increases endocortical osteoclast number in WT but not in a conditional KO mouse model deficient of Cx43 primarily in osteoblasts/osteocytes. Consistently, Cx43 deficiency reduces the induction of OC activity during unloading^23^ and hindlimb immobilization.^66^ Moreover, the trabecular OC surface shown in the study was increased in the R76W OVX group, which suggests the possible involvement of gap junctions in trabecular OC formation. Additionally, we could not exclude the potential synergistic effects with the impairment of both HCs and gap junctions observed in Δ130–136 mice.

Our cell culture studies showed that estrogen withdrawal decreased Cx43 levels and EtBr dye uptake. The decrease of dye uptake is possibly due to less HCs with similar activity or similar amount of HCs on cell surface with less activity. However, the open probability of HCs is very low under physiological conditions, and the activation of HCs is regulated by many factors, including mechanical stimulation, extracellular calcium concentration, plasma membrane voltage, protein–protein interactions, and redox status.^67^ Estrogen deficiency in MLO-Y4 cells has been reported to attenuate NO and PGE_2_ release and expression of DMP-1, SOST, and other bone-specific genes, thus diminishing osteocyte mechanosensitivity.^26^ Moreover, our earlier study shows that OS activates HCs in osteocytes.^68^ The opening of HCs mediates the release of factors, including ATP, PGEs, and other factors, from osteocytes.^13,17,69,70^ ATP can stimulate the formation and bone resorption activity of osteoclasts.^71^ Thus, ATP release is likely to be blocked by impaired HCs, consequently, attenuating the effect of ATP or other possible released factors on osteoclastogenesis and function. Together, besides its role in maintaining normal bone homeostasis, this study suggests a new mechanism involving Cx43 HCs in protecting osteocytes and bone tissue against cell death and catabolic effects, respectively, as a consequence of estrogen deficiency.

## Materials and methods

### Cell culture

MLO-Y4 cells were cultured on collagen-coated (rat tail collagen type I, BD Biosciences, 354236, 0.15 mg·mL^-1^) surfaces and were grown in phenol red-free α-minimum essential medium supplemented with 2.5% fetal bovine serum and 2.5% bovine calf serum, and incubated in a 5% CO_2_ incubator at 37 °C, as described previously.^72^

### Preparation of cell membrane extracts and Western blotting

Cultured cells were collected in lysis buffer (5 mmol·L^-1^ Tris, 5 mmol·L^-1^ EDTA/EGTA, and proteinase inhibitors) and then ruptured by pipetting through a 20 gauge needle. Cell lysates were then centrifuged at 45 000 × *g* for 45 min. The pellet was resuspended in lysis buffer and the membrane protein was dissolved by addition of sodium dodecyl sulfate (SDS) to a 1% final concentration. Protein concentrations of SDS-dissolved lysates were determined by Micro BCA Protein Kit (Thermo Scientific, Rockford, IL, USA) and the lysates were used for Western blotting analysis. Each protein sample was boiled in SDS loading buffer, subjected to electrophoresis on a 10% SDS-polyacrylamide gel, and electroblotted onto a nitrocellulose membrane. Membranes were incubated with a 1:300 dilution of affinity-purified anti-Cx43 antibody,^73^ or a 1:5 000 dilution of monoclonal anti-β-actin antibody (Sigma). Primary antibodies were detected with goat anti-rabbit IgG conjugated IRDye^®^ 800CW and goat anti-mouse IgG conjugated IRDye^®^ 680RD (1:15 000 dilution) using a Licor Odyssey Infrared Imager (Lincoln, NE, USA), as previously described.^74^ The band intensity was quantified by densitometry using Image J software (NIH, Bethesda, MD, USA).

### Dye uptake assay

Dye uptake analysis was performed as previously described.^75^ Briefly, MLO-Y4 cells were subjected to different treatments, and then washed three times for 5 min each with the recording solution (154 mmol·L^-1^ NaCl, 5.4 mmol·L^-1^ KCl, 1.8 mmol·L^-1^ CaCl_2_, 1 mmol·L^-1^ MgCl_2_,10 mmol·L^-1^ glucose, and 10 mmol·L^-1^ HEPES, pH 7.4). The cells were exposed to EtBr for 15 min, followed by rinsing three times with phosphate-buffered saline (PBS). Cells were fixed with 2% paraformaldehyde for 10 min and images were captured under a fluorescent microscope (BZ-X710, Keyence, Osaka, Japan). Image processing was performed off-line with ImageJ software (NIH, Bethesda, MD, USA). The collected data were illustrated as pixel mean in arbitrary units.

### Animal models and surgery procedure

We established two transgenic mouse models overexpressing dominant-negative Cx43 mutants, R76W and Δ130–136.^25^ We used a 17-week-old C57BL/6J WT and transgenic female mice (body weight about 19–24 g). The mice used in this study were all homozygous, and were bred separately. Ovariectomy or sham operations (as controls) were performed to model postmenopausal osteoporosis as described previously.^76^ Mice with specific genotypes were randomly assigned to experimental groups. All animal protocols were reviewed and approved by our Institutional Animal Care and Use Committee. Briefly, mice were anesthetized by intraperitoneal injection of 100 mg·kg^-1^ of ketamine (Butler Schein, Dublin, OH, USA) and 16 mg·kg^-1^ of xylazine (Butler Schein) prior to ovariectomy or sham operations. A 1 cm mid-dorsum incision was made to expose ovaries. The skin was gently separated from the underlying muscle using cotton swabs and ovaries were identified by white spots under the muscle on the flanks (the fat pad covering the ovary). Small incision over the white spots were made, and the ovaries with fat pads were removed by gently separating them from the uterine horn. The muscle and skin of the dorsum were sutured and one drop of 4% lidocaine was applied to the surgical site to minimize post-operative pain. The mice were housed in a temperature-controlled room with a light/dark cycle of 12 h at the UTHSCSA Institutional Lab Animal Research facility, under specific pathogen-free conditions. Food and water were provided ad libitum. All animal protocols were performed in accordance with the National Institutes of Health guidelines for care and use of laboratory animals. The animal protocols were approved by the UTHSCSA Institutional Animal Care and Use Committee.

### Isolation of RNA from bone tissues and real-time PCR

Real-time PCR was performed with total RNA isolated from humerus to detect the messenger RNA (mRNA) expression of SOD2. Long bone tissues (humerus) were isolated free of soft tissues, and bone marrow cavities were thoroughly flushed with PBS. The bone samples were then pulverized using a frozen mortar and pestle in liquid nitrogen. Total RNA was extracted using TriReagent (Molecular Research Center, Cincinnati, OH, USA) according to the manufacturer’s instructions. Real-time PCR was performed using an ABI 7900 PCR device (Life Technologies, Carlsbad, CA, USA) and SYBR Green (Life Technologies) with a two-step protocol (94 °C for 15 s and 64 °C for 60 s). The ΔΔC_T_ method was used for quantitative PCR data analysis. The primers of SOD2 are: sense, 5′-CAGATTGCTGCCTGCTCTAA-3′ and antisense, 5′-CTGAAGGTAGTAAGCGTGCTC-3′. Glyceraldehyde 3-phosphate dehydrogenase was used as a housekeeping gene control.

### BMD and μCT analysis

Mice were anesthetized by intraperitoneal injection of 100 mg·kg^-1^ of ketamine (Butler Schein, Dublin, OH, USA) and 16 mg·kg^-1^ of xylazine (Butler Schein). We used a dual-energy X-ray absorptiometry scanner, Lunar PIXImus Densitometer (GE Medical Systems, Piscataway, NJ, USA), to measure the BMD pre-operation and 1 month after ovariectomy surgery. The BMD value of the total body was acquired.

The OVX female mice were sedated under isoflurane (Baxter, Deerfield, IL, USA) and euthanized by cervical dislocation. Vertebrae and femurs from WT and transgenic mice were isolated. The structural properties of cortical and trabecular bones were evaluated using a 3D reconstructions of μCT imaging system (Brüker SkyScan 1173; Brüker microCT, Kontich, Belgium) as described previously.^25^ Samples were scanned in saline with the following settings: 60 kV, 167 mA beam intensity, 0.5 mm aluminum filter, 0.7° rotation step, 4-frame averaging, 1 090 ms integration time, 1 024 × 1 024 pixel matrix, and a 10 mm isotropic voxel dimension. After scanning, noise was removed from the images by eliminating disconnected objects smaller than 4 pixels in size. Two volumes of interest were selected in the L5 vertebrae and femur midshafts and automated contouring was used to delineate trabecular and cortical bone regions. In the L5 vertebrae, the trabecular bone volume of interest (VOI) was positioned 50 slices distal to the proximal growth plate and extended 150 slices in the distal direction. The VOI conformed to the endocortical boundary. An appropriate and uniform threshold was applied to all specimens after comparing grayscale and binarized images in both groups. For trabecular bone, a grayscale value of 80 in a set of 8-bit slices was set as the threshold. After thresholding, the BV/TV (%), Tb.Th (mm), Tb.Sp (mm), and Tb.N (mm^−1^) were quantified. Cortical bone structure was analyzed over 50 slices centered at the 55% of length (from proximal to distal) position in the femur diaphysis. We used the 55% length from the proximal end because of the low influence of the linea aspera on bone shape. This ridge is one of the insertion sites of the gluteal musculature and it contributes to an odd shape of the femur shaft. We treated all groups similarly. Grayscale values of 106 and 256 were set as the window for cortical bone. Cortical bone analyses included the diaphyseal total area (mm^2^), B.Ar (mm^2^), and the MMI.

### Mechanical testing of femur

Femurs were dissected from OVX WT and transgenic mice, and soft tissues were removed. Freshly isolated bones were kept frozen in saline-soaked gauze at −80 °C until three-point bending tests were performed. These tests were performed on an MTS Insight 5 Electromechanical system (MTS Systems Corporation, Eden Prairie, MN, USA) using Test Works software (version 4.0). The span distance for the three-point bending test was 8 mm and the loading pin was placed at the midshaft femur. The test was performed in displacement control mode at a constant rate of 0.5 mm·s^-1^ with data collected at a 200 Hz sampling rate for all measurements. Stress calculations were performed by taking into account the accurate cross-sectional areas and moments of inertia of each individual sample test span determined from µCT.

### Bone histology and histomorphometry

The femur and tibia bones isolated from OVX female mice were fixed in 4% paraformaldehyde for 2 days prior to decalcification with 10% EDTA (pH 7.5) for 3 weeks. The samples were embedded in paraffin, and 5-μm-thick sections were collected on glass slides and stained with H&E. The number of empty and total osteocytic lacunae was quantified. For TUNEL assay, an In Situ Cell Death Detection Kit, TMR red (Roche, Pleasanton, CA, USA) was used for detection and quantification of bone cell under apoptosis following the manufacturer’s instructions. Briefly, paraformaldehyde-fixed bone tissue sections were treated with proteinase K in Tris buffer containing 0.1% Triton X-100, and broken DNAs were labeled with TUNEL reaction mixture, which was prepared immediately before use by mixing Enzyme solution (terminal deoxynucleotidyl transferase) and Label solution (nucleotide mixture in reaction buffer). Tissue sections were analyzed and photographed using an Olympus IX70 microscope (Olympus, Japan), and the number of apoptotic cells was counted. Tissue sections were tartrate-resistant acid phosphatase stained using Leukocyte Acid Phosphatase Staining Kit (Sigma) according to the manufacturer’s protocols. NIH Image J was used to quantify Oc.S and BS in the metaphyseal trabecular bone and along the endocortical surface from the metaphysis to the upper midshaft. The Oc.S/BS value was then calculated. To measure the number of osteoblasts, we used modified Masson’s trichrome Staining. Briefly, sections were stained with Harris hematoxylin to visualize nuclei, acid Fuchsin-Ponceau to stain osteoid, and finally Toluidine blue to better visualize osteoblast morphology and to distinguish mineralized bone from osteoid.

### Immunohistochemistry

We used anti-4-HNE and Cx43 immunohistochemistry staining to assess the OS-induced lipid peroxidation and Cx43 expression. The ABC (avidin–biotin–peroxidase complex) Immunostaining Assay Kit (Vector Laboratories, PK-6101) was used as previously reported.^77^ Briefly, bone tissue sections were antigen unmasked using sodium citrate buffer (pH 6.0) at 65 °C for 2 h for 4-HNE staining and trypsin digestion buffer (pH 7.8) at 37 °C for 30 min for Cx43 staining. Bone tissue sections were then treated with rabbit normal serum for 20 min at room temperature to block nonspecific background staining. Tissue sections were stained with anti-4-HNE monoclonal antibody (1:75 dilution, Abcam, ab46545) for 30 min at room temperature, or labeled with 1:50 dilution of affinity-purified Cx43 antibody^73^ at 4 °C overnight. Then, the sections were incubated for 30 min with biotin-labeled secondary antibody and VECTASTAIN ABC Reagent for 30 min. Samples were washed in PBS buffer and developed in DAB (SK4100) chromogen solution (Vector Laboratories, Burlingame, CA, USA). Tissues were then counterstained with VECTOR Hematoxylin (H-3401) for 5 min at room temperature and mounted. Sections were photographed using the Keyence microscope (BZ-X710, Keyence, Osaka, Japan). The numbers of 4-HNE-positive signals per mm^2^ were quantified using NIH Image J software. The Cx43 immunoreactive area and integrated optical density (IOD) were quantified by Image Pro Plus software (Media Cybernetics, Silver Spring, MD, USA), and the ratio of IOD (average Cx43 staining intensity) was determined by IOD normalized to the corresponding area.

### Calcein and alizarin labeling, and dynamic bone histomorphometry

Two weeks after surgery, the mice were subjected to an intraperitoneal injection of calcein (Sigma) at 30 mg·kg^-1^ body weight, followed by an alizarin (Sigma) injection 10 days later. Four days after the second injection, the mice were euthanized, and femurs and tibias were dissected and embedded in undecalcified methylmethacrylate for plastic tissue sectioning. The embedded samples were cut to 8 µm tissue sections by a skiving machine (Leica RM2265). Digital images were obtained using a fluorescence microscope (Olympus). The following bone parameters were quantified in the trabecular bone and endocortical regions with Image J software: total perimeter (BS); single label perimeter (sLS); double label perimeter (dLS), and distance between labels (Ir.L.Th). The following values were then calculated: mineralizing surface [MS/BS = (sLS/2 + dLS)/BS], mineral apposition rate (MAR = Ir.L.Th/10), and bone formation rate (BFR/BS = MAR*(sLS/2 + dLS)/BS).

### Statistical analysis

Statistical analysis was performed using GraphPad Prism5 statistics software (GraphPad). All data are presented as mean ± SEM. *T* test and two-way analysis of variance with Tukey’s test was used for statistical analysis. Asterisks indicate the degree of significant differences compared with the controls (*P < 0.05; **P < 0.01; ***P < 0.001).

## Supplementary information

Supplemental figures-marked up
