# Supplementary material for: Connexin 43 hemichannels protect bone loss during estrogen deficiency
Source: Bone Res. 2019 Apr 22;7:11. doi: 10.1038/s41413-019-0050-2 (PMC6476886; doi:10.1038/s41413-019-0050-2)

Figure S1. Estrogen treatment for 24 hours increased Cx43 expression without affecting EtBr dye uptake in MLO-Y4 cells.

(A) Membrane extracts were subjected to immunoblotting using Cx43 (CT) or β-actin antibodies. The right panel shows the densitometric measurement ratios of Cx43 to β-actin (n = 3). (B) Hemichannel dye uptake was performed with EtBr (red fluorescence). Left panels showed representative fluorescence images of treated MLO-Y4 cells after dye uptake. The intensity of EtBr fluorescence was measured and quantified (right panel). Data shown are mean ± SEM. *, $P < 0.05$; **, $P < 0.01$.

Figure S2. Ovariectomy induced changes of body weight, uterus weight and BMD in WT and transgenic mice. (A) Increased body weight and decreased uterus weight in WT OVX mice compared to sham group 4 weeks after surgery. Scale bar, 5 mm. **(B)** BMD of total bones was measured by DXA analysis 4 weeks after ovariectomy of WT and transgenic mice. Left panels show the corresponding percentage changes. Data shown are mean ± SEM. *, $P < 0.05$, **, $P < 0.01$, ***, $P<0.001$. n = 5-8.

Figure S3. Minimal effect of ovariectomy on femoral trabecular bones of WT and transgenic mice. 3D μCT analyses of BV/TV (A), Tb.N (B), Tb.Th (C) and Tb.Sp (D) of femoral trabecular bone were performed. Significant bone loss was observed in WT and R76W, but not in Δ130-136 mice (A), while significant loss of Tb.N. was shown in WT and two transgenic mice (B). No significant changes of Tb.Th. (C) and Tb.Sp (D) except WT. Left panels show the corresponding percentage changes. Data shown are mean ± SEM. *, $P < 0.05$. n = 6-7. BV/TV, bone volume fraction; Tb.N, trabecular number; Tb.Th, trabecular thickness; Tb.Sp, trabecular spacing.

Figure S4. The size of cortical bone marrow cavity area was not affected by ovariectomy in WT and transgenic mice. μCT analysis of femur cortical bone marrow area showed no difference in WT and transgenic groups after ovariectomy. Data shown are mean ± SEM. n = 4-7.

Figure S5. Apoptotic osteocytes in trabecular bone showed trend of increase in Δ130-136 mice after ovariectomy. Tibia trabecular osteocyte apoptosis was analyzed using TUNEL staining. Δ130-136 OVX mice exhibited a near significant increase ($p = 0.07$) of osteocyte apoptosis compared to sham. TUNEL signals per mm^2 of trabecular bone area were quantified by NIH Image J. Data shown are mean ± SEM. n = 3-6.

Fig.S1

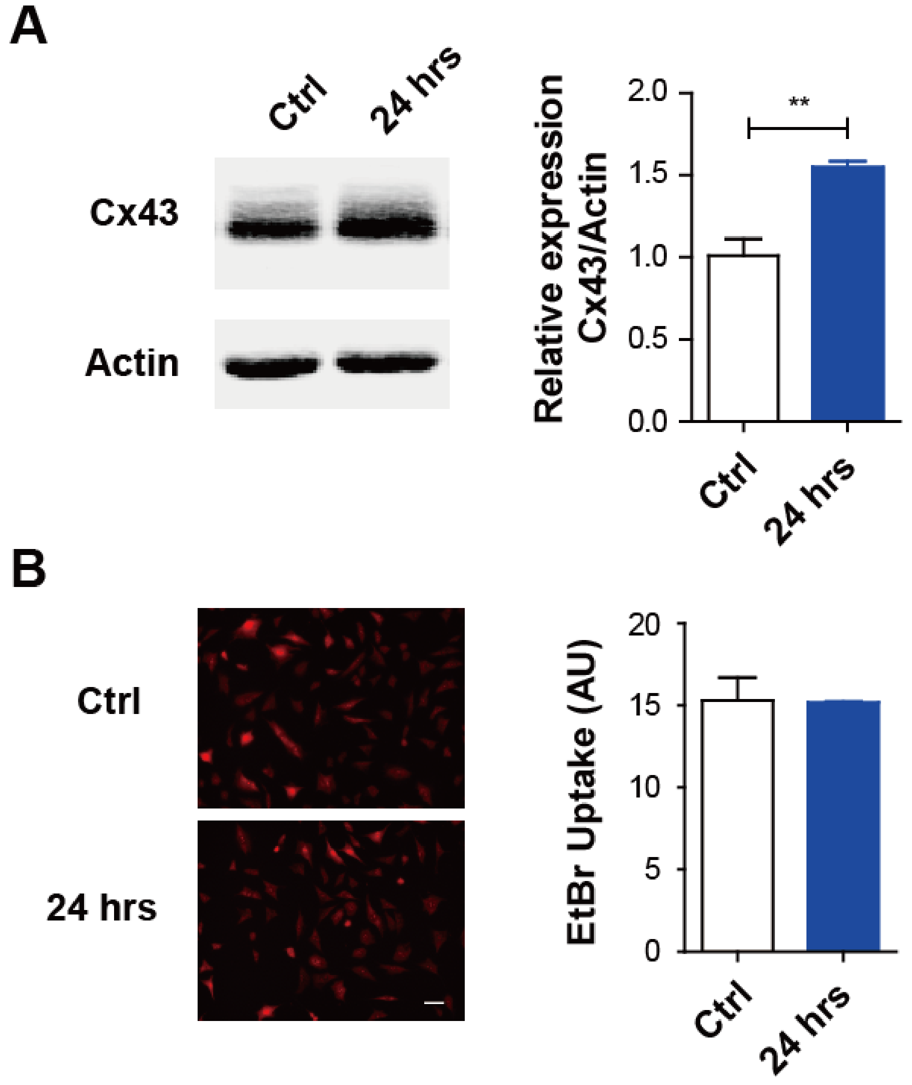
A
Ctrl
24 hrs
Cx43
Actin
Relative expression Cx43/Actin
2.0
1.5
1.0
0.5
0.0
**
Ctrl
24 hrs
B
Ctrl
24 hrs
EtBr Uptake (AU)
20
15
10
5
0
Ctrl
24 hrs

Fig.S2

A

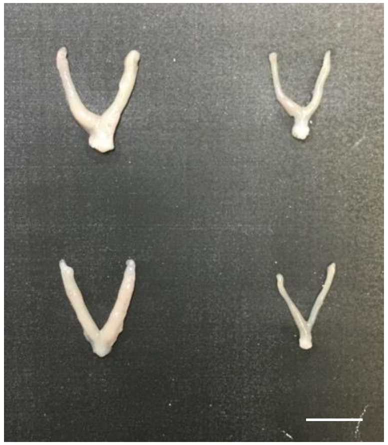

	Body weight before surgery (g)	Body weight at sacrifice (g)	Uterus weight (mg)
WT Sham	22.37±0.49	22.64±0.46	89.86±14.65
WT OVX	22.30±0.31	24.81±0.49**	19.75±10.54***

B

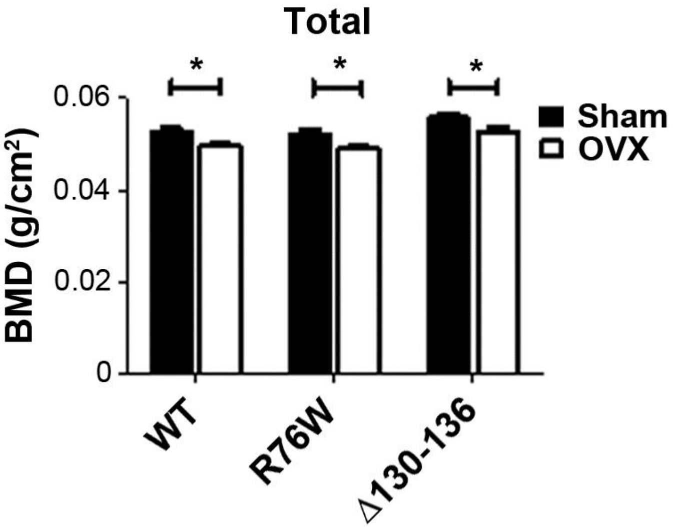

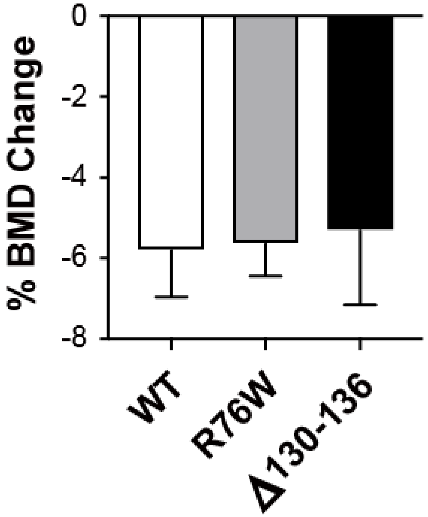

Fig.S3

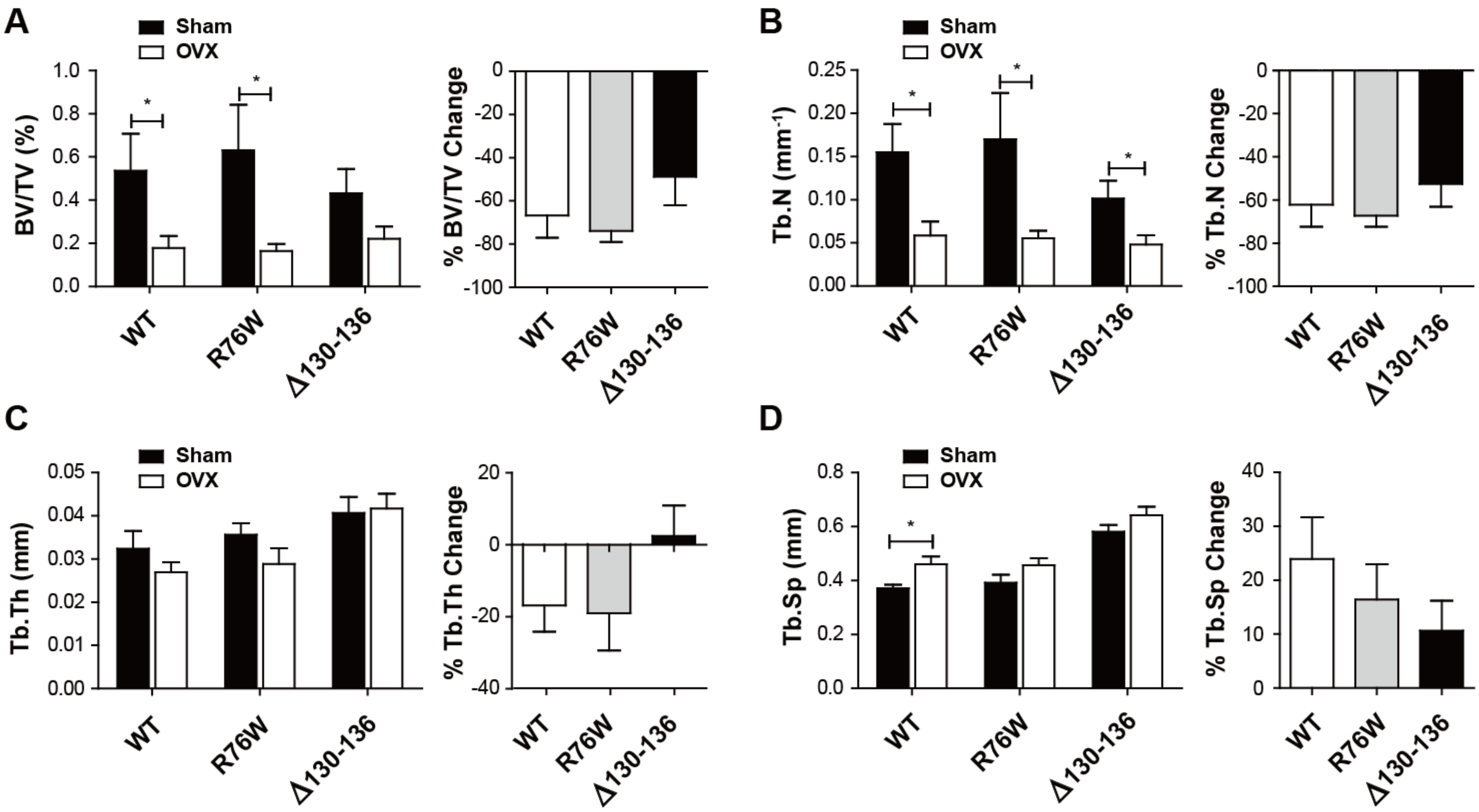
A
Sham
OVX
BV/TV (%)
1.0
0.8
0.6
0.4
0.2
0.0
*
WT
R76W
Δ130-136
% BV/TV Change
0
-20
-40
-60
-80
-100
B
Tb.N (mm-1)
0.25
0.20
0.15
0.10
0.05
0.00
% Tb.N Change
C
Tb.Th (mm)
0.05
0.04
0.03
0.02
0.01
0.00
% Tb.Th Change
20
0
-20
-40
D
Tb.Sp (mm)
0.8
0.6
0.4
0.2
0.0
% Tb.Sp Change
40
30
20
10
0

Fig.S4

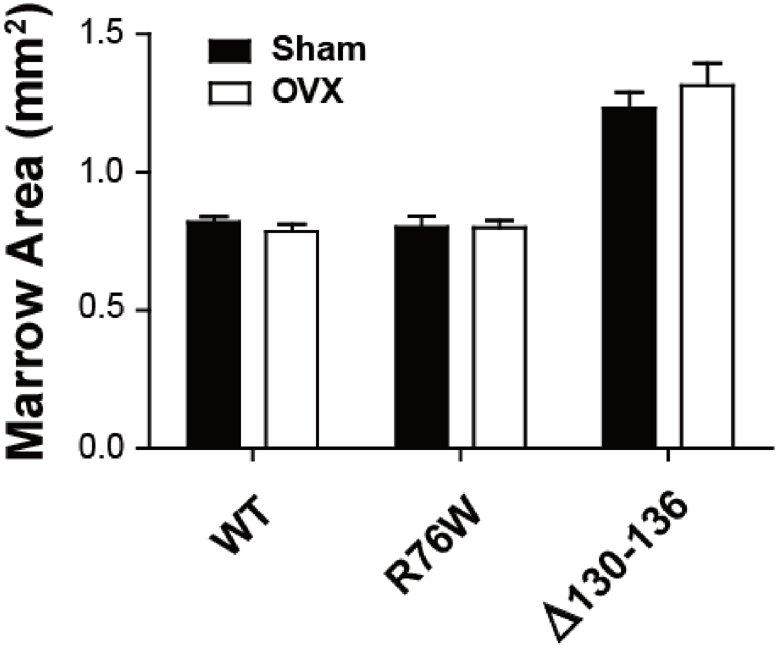

Fig.S5

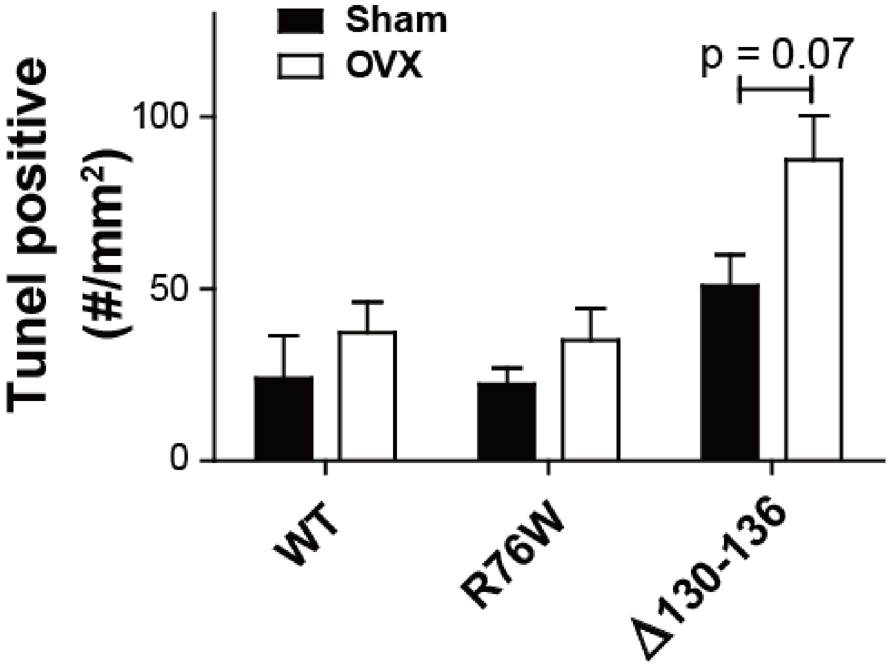

Supplement: Supplementary file 1 — Supplemental figures-marked up [file 41413_2019_50_MOESM1_ESM.pdf]
